# Gas phase polymerization of ethylene towards UHMWPE

**DOI:** 10.3906/kim-1907-48

**Published:** 2020-06-01

**Authors:** Gözde GEÇİM, Ertuğrul ERKOÇ

**Affiliations:** 1 1Department of Chemical Engineering, Faculty of Engineering and Natural Sciences, Bursa Technical University, Bursa Turkey; 2 Admire-Tech Inc., Bursa Turkey

**Keywords:** Gas phase polymerization, Taguchi experimental design, UHMWPE, Ziegler-Natta catalysts

## Abstract

For the first time, ultra-high molecular weight polyethylene (UHMWPE) was produced in gas phase process with a new fluidized bed concept where the solids are dispersed phase and the gas is bulk phase as opposed to conventional fluidized bed reactors (FBRs). With this concept, UHMWPE with average molecular weights about 1-6,9 × 10^6^ g mole^-1^ were produced with a commercial supported Ziegler-Natta catalyst by using a gas phase mini semibatch reactor system. Additionally, optimum conditions of gas phase polymerization for the best results of productivity, catalyst activity, molecular weight and crystallinity were determined by Taguchi experimental design and catalyst stability at the optimum condition was tested by video microscopy polymerization. The characterization of products was carried out experimentally by TGA, DSC, FTIR, and NMR.

## 1. Introduction

Although HDPE can be produced both by fluidized bed and slurry technologies, currently UHMWPE is produced by only slurry process. Considering the high capital investment for ethylene production and the relatively cheap prices of polyethylene, fluidized bed reactor technology offers substantial advantages to reduce the capital and operational costs. Since the olefin polymerization reactions are strongly exothermic with 293 J g−1 [1], an efficient heat transfer is extremely important. To maintain a uniform temperature profile over the catalysts, in UHMWPE process, slurry reactors are used where solvent is added to the reactor to remove the heat from the solid catalyst surface. However, the added liquid phase contributes to a gas-liquid mass transfer limitation which can cause a reduction in reaction conversion [2] and the separation of the solvent from the polymer is rather expensive in terms of both construction and operation [3]. Moreover, to increase the mass transfer from gas to liquid, the pressure should be increased. In Fluidized Beds however, since there is no limitation in mass transfer [4], the reactor can be operated in lower pressures [5], and having no solvents, offers an easier and cheaper operation. While to use FBR technology seems more advantageous over slurry reactor technology, one can wonder why FBR technology does not take its place in UHMWPE process. The underlying reason for this can be the complicated hydrodynamics of fluidized beds which results in poor heat transfer, formation of hotspots and consequently, catalyst poisoning. Similar to slurry reactors, FBRs also use gas as a dispersed phase. In both technologies the gas is mostly distributed from a gas sparger, a unit that affects the hydrodynamics. Bubbles move along the bed, but because of the hydrodynamics, they breakup or coalescence. Especially for olefin polymerization reactions, where the catalyst is very sensitive to temperature, the complicated hydrodynamics of the fluidized bed causes oscillations in temperatures [6] which deteriorate the process significantly. As ethylene polymerization takes place in bubbly flow, the effect of particle size distribution plays important role. Especially Ziegler-Natta catalysts can have sizes of 20 μm, falls into the Geldart Group C powders and these type of solids are the most difficult to fluidize [7]. Additionally, while the reactor starts the operation in Geldart C, as the formation of polymer takes place, the classification, and hence the behavior of the particles and reactor dynamics also change. To overcome the complexities of nonideal fluidized bed reactors, the researchers try to eliminate the disadvantages by reshaping the structure of FBRs [8], determine the rules to scale up [for a review see (Rüdisüli et al., 2012)] and apply several process intensification techniques [for a review see (Zhang, 2009)] [9,10]. If these challenges were met, the fluidized bed technology can be used for UHMWPE process.

Here, we suggest a different strategy to use gas-solid system, a fluidized bed that diverts fundamentally from the classical approach. In the classical approach, the gas is distributed, that is the gas is the dispersed phase and the solids are the bulk phase. In order to achieve efficient heat and mass transfer rates, without having hot spots, we suggest to distribute the solids in the gas that is the solids are the dispersed phase and the gas is the bulk phase. In this configuration, the bubbles do not exist and do not cause any complexity in the hydrodynamics, and the gas phase is turbulent enough to have a homogenous, well mixed phase, i.e., the particles move together with the gas no matter what their sizes are, thereby to have only emulsion phase inside the reactor. While there are no studies of UHMWPE synthesis in gas phase, most of the attention is given to new catalyst designs, but since this study is performed with commercial Ziegler-Natta catalyst and catalyst design is out of scope, the reader is referred to [11–16].

Since there is no reported gas phase process for the production of UHMWPE, to have a better understanding of the process, a short state of the art for the gas phase process of high density polyethylene (HDPE) is given. Temperature and pressure are the most important parameters which affect the molecular weight and polymerization activity. While the increase in temperature decreases the molecular weight of the polymer due to the increase of chain transfer more than chain propagation, molecular weight increases with pressure depending on the increase in the ethylene insertion [17]. For the polymerization activity, temperature was found to be the most important factor, and has an important relation with pressure and cocatalyst. Increasing the pressure decreases the polymerization activity, however, cocatalyst has no considerable effect on the activity [18]. Ethylene feed rate is another important parameter and reported to cause thermal runaway and catalyst deactivation when exceeds its maximum value [19].

In this work, new approach in fluidized bed technology was applied to realize gas phase production of this unique polymer.

## 2. Materials and methods

### 2.1. Materials

Ethylene and argon gases were obtained from Linde Industrial Gases (Linde Public Limited Company, Surrey, UK) with the purities of 99.9% and 99.9996%, respectively. A commercial Ziegler-Natta catalyst supplied by Petkim Petrokimya Holding A.Ş. (İzmir, Turkey) (10% w/w) with a titanium compound of about 78 mg g^-1^ catalyst was used. Triethylaluminum (TEA) cocatalyst was purchased from Alfa Aesar [Thermo Fisher (Kandel) GmbH, Kandel, Germany]. Monomer gas was purified with Molecular sieve 3A°(Alfa Aesar) and CaCl_2_ (Merck KGaA, Darmstadt, Germany). Decahydronaphtaline (Alfa Aesar) was used as solvent.

### 2.2. Experimental method

**Figure 1:**
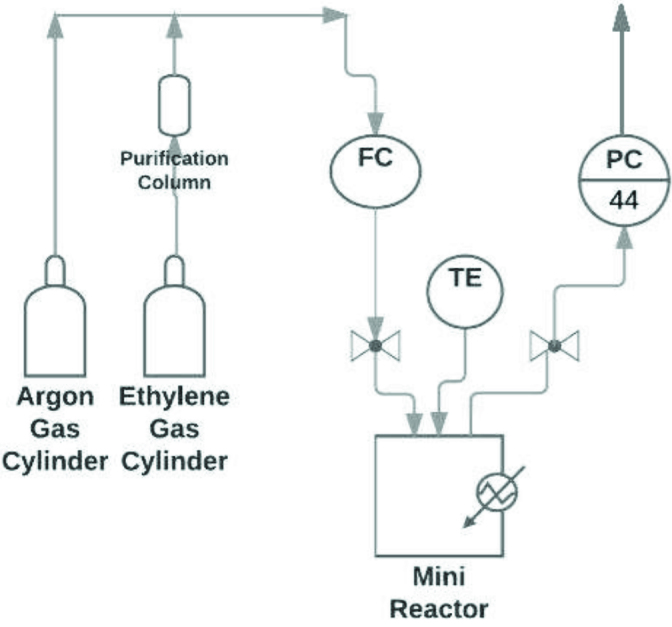
Experimental setup. FC: Flow controller, PC: Pressure controller, TE: Thermocouple.

A stainless steel mini reactor with 0.08 L volume was used for the polymerization reactions. The reaction system shown in Figure 1 was used for the experiments. First all the lines were vacuumed followed by purging with argon gas to remove oxygen and humidity from the system. The ethylene gas was purified using columns filled by molecular sieve 3A° and CaCl2 before being fed to the reactor. The flow rate of ethylene gas was controlled by ethylene mass flow controller (Bronkhorst F-201-CM), and the pressure inside the reactor was controlled by a back pressure controller (Bronkhorst P-702-CM) connected to the outlet of the reactor. The experiments were performed under strongly turbulent flow regime with varying pressures and temperatures.

### 2.3. Experimental design

The main parameters that affect the molecular weight are reaction temperature, pressure, TEA/Ti ratio and reaction time. With 4 main parameters, an experimental design is necessary, and 4 main levels were chosen for each parameter as shown in Table 1.

**Table 1:** Parameters and their levels for experimental design.

Parameters	Unit	Level 1	Level 2	Level 3	Level 4
A temperature	°C	30	45	60	75
B pressure	bars	3	7	10	15
C TEA/Ti	-	1	2	2.2	2.5
D time	min	30	60	90	120

Taguchi optimization method [20,21,22] was applied to decrease experiment number and analyze the effects of these parameters, efficiently. L_16_ (4^4^) orthogonal array was chosen for these parameters and affected results, which is 16 experiments to run in total. Additional 4 experiments were run for each of the optimum conditions gathered to validate the optimum values. While the productivity (g PE g catalyst^-1^ h^-1^) and catalyst activity (g PE g catalyst^-1^) values should be as high as possible, crystallinity (%) and molecular weight (g moles^-1^) values should be between 39%–75% and 3–10 × 10^6^ g moles^-1^ , respectively. “Larger is better” approach was performed and the highest value of each result was determined as optimum level according to the signal/noise (S/N) mean plots calculated from Eq (1). S/N value measures the variation of process conditions which should be stable and shows the maximum mean value of factor levels for each parameter [23].

(1)S/N=-10log[meanstandard deviation]=-10log[1η∑i=1η1γi2]

where η is the number of repetition, y_i_ is the performance value of the i th experiment.

Analysis of variance (ANOVA) was applied to evaluate the confidence levels of results and how they were affected by selected parameters [23,24,25]. ANOVA table typically includes sum of squares of factors and total, variance and error values of variance were calculated as follows:

*Degrees of freedom:*

(2)DFF=L-1

*Error variance:*

(3)σε2=SSErrorDFError

*Percentage contribution:*

(4)PF=SSFSSTx100

m is the average of sixteen ηi values, L means level.

(5)Yi=+Xi+ei

Y_i_ is the predicted value, μis the overall mean, X_i_ is the sum of the effects of the 4 factors, e_i_ random error for experiment i.

(6)Se=±2[1n0}σε2+[1nr]σε2

(7)1n0=1n+[1nAi-1n]+[1nBi-1n]+[1nCi-1n]+[1nDi-1n]

S_e_ is the confidence limit which is attached to and subtracted from predicted value to determine confidence interval.

In addition, for the experimental results in percentage (%), Ω transformation was performed to evaluate predicted value and then reverse transformation to the experimental results was performed for confidence limit value:

(8)Ω(db)=-10log (1P-1).

### 2.4. Polymer characterization

Since the obtained polymers were expected to have relatively high molecular weight, the intrinsic viscosity of the polymers was measured with an Ubbelohde viscometer and viscosity-average molecular weight (Mv) of polymers was calculated by using Mark-Houwink-Sakurada equation [26]. First, the flow time of decalin used as the solvent was measured and then the flow times of polymer solutions in different concentrations (0.01-0.07 g dL^-1^) were measured at 135 °C. Molecular weight of the polymers was determined according to formulas given below [26].

Relative viscosity;

(9)ηr=tsolutiontsolvent

Specific viscosity;

(10)ηsp=ηr-1,

Intrinsic Viscosity;

(11)ηspc=[η].

The calculated intrinsic viscosity values were used for molecular weight determination by using Mark-Houwink-Sakurada equation.

(12)[η]=KMνα

K is 6.2 × 10^-4^ and α is 0.7 for polyethylene [27].

The purity of the samples and the presence of terminal vinyl groups were determined with a Thermo Scientific Nicolet iS50 FT-IR spectrometer (Thermo Fisher Scientific Inc., Waltham, MA, USA) at a wavelength range of 500–4000 cm^-1^ and at a resolution of 4 cm^-1^. The terminal vinyl groups and branched vinyl groups are expected to show vibrations at about 910 cm^-1^ and 965 cm^-1^, respectively [28].

The crystallization degree of the produced polymers was determined by using the theoretical value of enthalpy of fusion (293 J g^-1^) [1] with a PerkinElmer DSC 8000 (PerkinElmer Corporation, Waltham, MA USA). DSC thermogram was obtained by heating the sample from 50 °C to 180 °C with a heating rate of 10 °C min^-1^, then cooling the sample until 50 °C with a cooling rate of 10 °C min^-1^, and then heating again until 180 °C with the same heating rate in the first stage under nitrogen atmosphere. By running a second heating ramp, thermal history of the produced polymers was eliminated. The crystallinity degree, X (%) was calculated according to the below formula [29].

(13)X=(ΔH/293)x1000

In order to determine the decomposition temperature of the produced polymers and the amount of inorganic catalyst residuals, TGA (PerkinElmer STA 6000) analysis was applied. The temperature of polymer was ramped from 30 °C to 600 °C with a heating rate of 20 °C min^-1^ under nitrogen atmosphere.

NMR experiments were performed on a Bruker Superconducting FT. NMR Spectrometer, operating at a ^13^C 8500 Hz spin rate with 4 mm MAS (Magic Angle Spinning) probe. Determination of the produced polymer morphology in terms of crystalline and amorphous regions was achieved by the interpretation of NMR spectra.

### 2.5. Catalyst stability for optimum conditions

Video microscopy method was used to observe the activity of the catalyst particles during polymerization. Leica Microscope (Leica Microsystems GmbH, Wetzlar, Germany) was used with an objective type of 10 × /0.25, and images were taken in every 0,1 s (10 frames/s) for the first 2.5 min of polymerization, then every 1 second (1 frame/s) for the remaining polymerization time. Rate of polymerization (Rp) was determined by calculating equivalent area (EA), equivalent circle diameter (ECD) and equivalent sphere volume (ESV) from images analyzed with Shadow Sizing module of Dynamic Studio program®[30].

(14)ECD=4xEAπ2

(15)ESV=ECD3xπ6

(16)Rp=(ESVt-ESVo)xρPEESVoxρCatx(1-Φ)xdt

Where Rp is the rate of polymerization produced mass of polymer per gram catalyst per h, dt is the polymerization time, ∅ is the porosity of the catalyst, ρ_PE_ and ρ_Cat_ are the densities of polyethylene and catalyst, respectively.

## 3. Results and discussion

### 3.1. Experimental results

Maintaining the strong turbulence, the experiments were carried out with Reynolds number of around 280,000. The experiments designed by Taguchi experimental design and results are shown in Table 2. The results show that UHMWPE with molecular weights greater than 3x10^6^ g moles^-1^ were produced successfully for many cases.

**Table 2:** Experimental results.

Exp.	A	B	C	D	Cat. act.^a^	Productivity^b^	X^c^	IV^d^	M_v_×10^6(e)^
1	30	3	1	30	8.18	16.36	40.52	7.26	1.02
2	30	7	2	60	16.19	16.19	43.43	21.56	5.18
3	30	10	2.2	90	35.84	23.89	41.28	12.78	2.38
4	30	15	2.5	120	12.71	6.36	40.69	13.84	2.68
5	45	3	2.2	60	42.20	42.20	47.12	8.87	1.38
6	45	7	2.5	30	26.87	53.74	44.60	18.64	4.17
7	45	10	1	120	23.88	11.94	41.88	26.11	6.89
8	45	15	2	90	44.48	29.65	46.70	21.11	5.02
9	60	3	2.5	90	25.00	16.67	53.64	13.55	2.59
10	60	7	2.2	120	69.51	34.75	57.24	18.25	4.04
11	60	10	2	30	12.58	25.16	44.47	9.80	1.60
12	60	15	1	60	18.51	18.51	43.16	21.94	5.32
13	75	3	2	120	17.23	8.62	50.68	6.71	0.91
14	75	7	1	90	15.31	10.21	50.01	24.75	6.37
15	75	10	2.5	60	12.69	12.69	52.25	9.48	1.52
16	75	15	2.2	30	12.87	39.42	50.24	3.12	0.29

^a^g PE g catalyst^-1^; ^b^g PE g catalyst^-1^ h^-1^; ^c^X: Crystallinity degree (%) ^d^IV: Intrinsic viscosity (dlg^-1^); ^e^Mv: Viscosity-average molecular weight (g moles^-1^).

**Figure 2:**
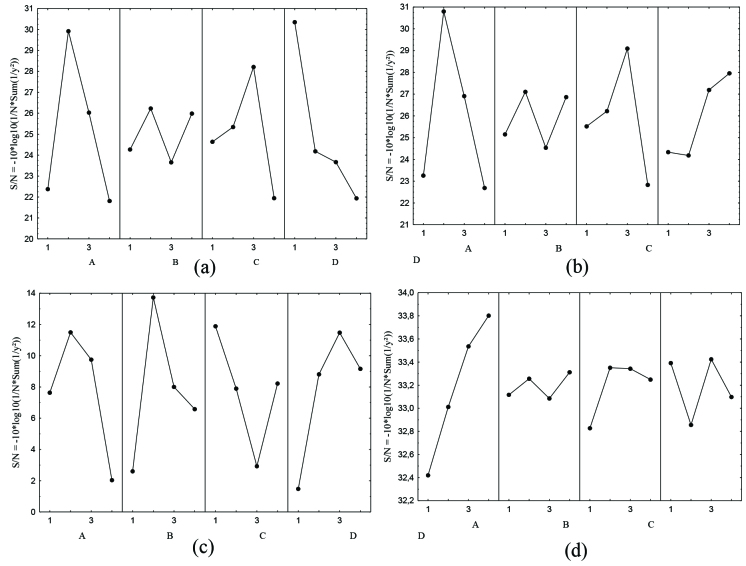
Optimum working conditions a) Productivity (O1) b) Catalyst activity (O2) c) Molecular weight (O3) d) Crystallinity (O4).

The important outputs of UHMWPE production is the highest molecular weight and crystallinity values. The mean value of the S/N ratio in decibel (dB) at 4 levels of the process parameter for A (temperature, °C), B (pressure, bars), C (TEA/Ti) and D (polymerization time, min) were demonstrated in Figures 2a–2d, respectively. According to the S/N plots, optimum conditions were given in Table 3. Having 4 observed quantities namely productivity (1), catalyst activity (2), molecular weight (3) and crystallinity (4), there are 4 optimum values for each, O1, O2, O3 and O4 respectively.

**Table 3:** Optimum conditions determined by experimental design.

Experiment	A (T°C)	B (P-bars)	C (TEA/Ti)	D (Time-min)
O1	45	7	2.2	30
O2	45	7	2.2	120
O3	45	7	1	90
O4	75	15	2	90

Figure 2a illustrates the effect of parameters on productivity. Optimum conditions were 45 °C, 7 bars, 2.2 for TEA/Ti and 30 min of reaction time (O1). The productivity in the process increased sharply with the increase in temperature, pressure and TEA/Ti ratio until the optimal levels were reached. The increase in pressure resulted in increase of ethylene monomer amount in the polymerization media. However, increasing the temperature and TEA/Ti ratio further, caused decrease in the productivity of the polymerization. Figure 2b depicts the effects of parameters on catalyst activity. A similar behavior was observed concerning temperature, pressure and TEA/Ti ratios, while for polymerization time optimum value was 120 min and catalyst activityincreased with increasing polymerization time. Consequently, the optimum parameters for the catalyst activity (O2) were 45 °C, 7 bars, 2.2 for TEA/Ti and 30 min of reaction time. Optimum conditions for molecular weight were 45 °C, 7 bars, 1 for TEA/Ti and 90 min of reaction time (O3), as shown in Figure 2c. While the increase in temperature, pressure and polymerization time resulted in higher molecular weights, higher values of TEA/Ti ratio showed adverse effect, due to the chain termination caused by transfer of excess aluminum available in TEA. The crystallinity of polymers increased with increasing temperature as shown in Figure 2d, because ofthe positive effect of temperature on the orientation of chain structure inside the polymer and so the optimum values for crystallinity were 75 °C, 15 bars, 2 for TEA/Ti and 90 min of reaction time (O4).

To determine the effects of parameters on process results, 4 additional experiments were run, 1 experiment for 1 optimum condition (at the conditions of O1, O2, O3, and O4), ANOVA was used and confidence limits of the optimum results were presented in the Table 4. F test (Fischer ratio) was performed in order to understand the most dominant factor on experimental results [31]. It is evident from Table 4 that polymerization time and temperature were the most effective parameters for productivity. The error value of variance was used to determine confidence limits of this output represented also in the Table 4. Despite the fact that productivity and catalyst activity had similar optimum values for process parameters, the effects of the parameters differ from each other in their magnitudes. The biggest effect was introduced by temperature. The observed value of the crystallinity remained between the confidence interval of the design for catalyst activity (Table 4). The highest F-value was obtained by second parameter which was the monomer pressure, which shows that the most effective parameter in molecular weight determination is the process pressure. On the contrary of other process outputs, all of the parameters significantly affected the molecular weight shown by the higher F-values than critical F-value. Temperature was the most effective parameter for crystallinity as indicated in the S/N plots (Figure 2d) and what’s more the observed value of crystallinity for optimum conditions was in the range of confidence limits.

**Table 4:** ANOVA table for S/N ratio.

Factor	SS^a^	DOF^b^	V	F	PC^c^	PV^d^	CI^e^	OV^f^
*Productivity*								
A	1183.53	3	394.51	6.70	42.28	61.51	43.92–79.09	53.74
B	129.30	3	43.10	0.73	4.62			
C	214.41	3	71.47	1.21	7.66			
D	1272.38	3	424.13	7.20	45.45			
Error	176.72	3	58.91					
*Cat. act.*								
A	1457.11	3	485.70	14.08	56.70	34.46	21.01–47.92	45.92
B	79.64	3	26.55	0.77	3.09			
C	571.41	3	190.47	5.52	22.23			
D	461.91	3	153.97	4.46	17.97			
Error	103.50	3	34.50					
*Mv*								
A	9.60	3	3.20	5.78	15.04	4.58	2.57–6.58	4.17
B	24.12	3	8.04	14.51	37.77			
C	17.95	3	5.98	10.80	28.12			
D	12.18	3	4.06	7.33	19.07			
Error	1.66	3	0.55					
*Crystallinity*								
A	130.43	3	43.48	7.36	71.81	51.60	46.03–57.17	48.68
B	4.11	3	1.37	0.23	2.26			
C	25.73	3	8.58	1.45	14.17			
D	21.37	3	7.12	1.21	11.77			
Error	17.73	3	5.91					

^a^SS: Sum of squares; ^b^DOF: Degree of freedom; ^c^PC: Percentage contribution; ^d^PV: Predicted value; ^e^CI: Confidence interval; ^f^OV: Observed value.

### 3.2. Polymer characterization

**Figure 3:**
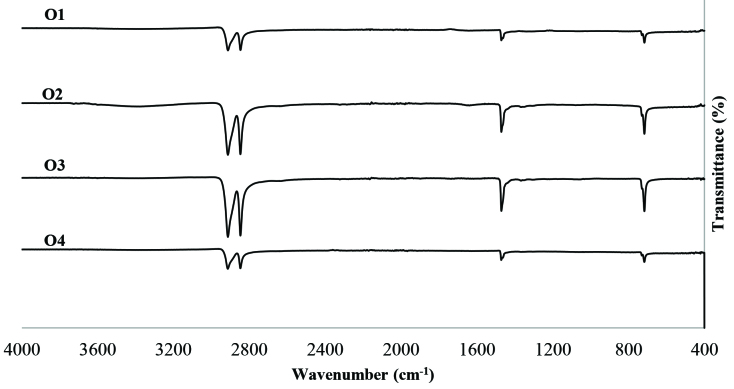
FTIR results for optimum conditions. O1: Optimum experiment for productivity; O2: Optimum experiment for catalyst activity; O3: Optimum experiment for molecular weight; O4: Optimum experiment for crystallinity.

FTIR spectra of the produced polymers at the optimum conditions (O1 to O4) are shown in Figure 3. Absorption bands for terminal vinyl band stretching at 910 cm^-1^ and terminal branched vinyl group band stretching at 965 cm^-1^ were not observed indicating that the produced polymers do not contain any unreacted monomer or impurities. Peaks around 2913–2850 cm ^-1^ correspond to asymmetric and symmetric stretching of CH_2_, 1470 cm^-1^ corresponds to bending vibration and 716 cm−1 corresponds to wagging deformation.

**Figure 4:**
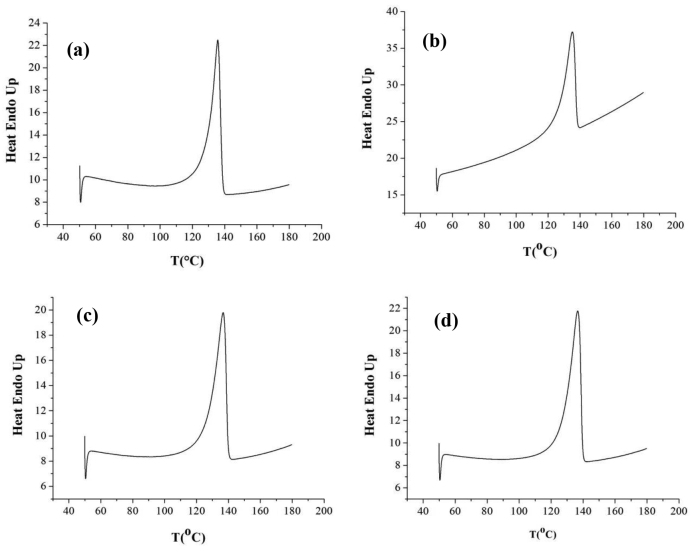
DSC Analysis for a) Productivity (O1) b) Catalyst activity (O2) c) Molecular weight (O3) d) Crystallinity (O4).

T_m_ values of the produced polymers were determined by assigning the maximum peak of the endotherms. According to DSC thermogram shown in Figure 4, melting point of the produced polymer in optimum value O1 was about 130–145 o C, high enough confirming a UHMWPE crystal. In general, LDPE and LLDPE show melting points of about 98–115 °C and 100–125 °C, respectively [32]. On the other hand, UHMWPE exhibits a sharper incipient melting that shows the crystallinity difference between chains is smaller. Because of the long chains, melting point of polymer increases, conformation of chains gets harder and an extra small peak appears in the melting line due to crystal gaps. A second cooling-heating cycle was run to eliminate the effect of processing conditions and to perfect the crystals. The percent crystallinity of the produced polymers O1, O2, O3 and O4 was calculated from DSC curve integration as 44,94%, 47,24%, 46,91%, and 48,58%, respectively (Figure 4).

**Figure 5:**
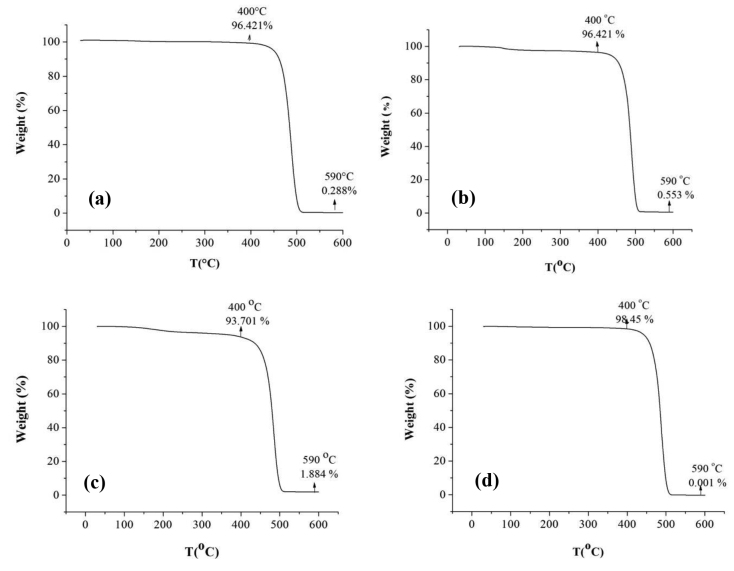
TGA thermogram for a) Productivity (O1) b) Catalyst activity (O2) c) Molecular weight (O3) d) Crystallinity (O4).

In the thermogram of polymers (Figure 5), it was observed that 90–98% of the samples decomposed. The rest of the mass which remained very low by comparison with entire mass was determined as inorganic residual of catalyst.

**Figure 6:**
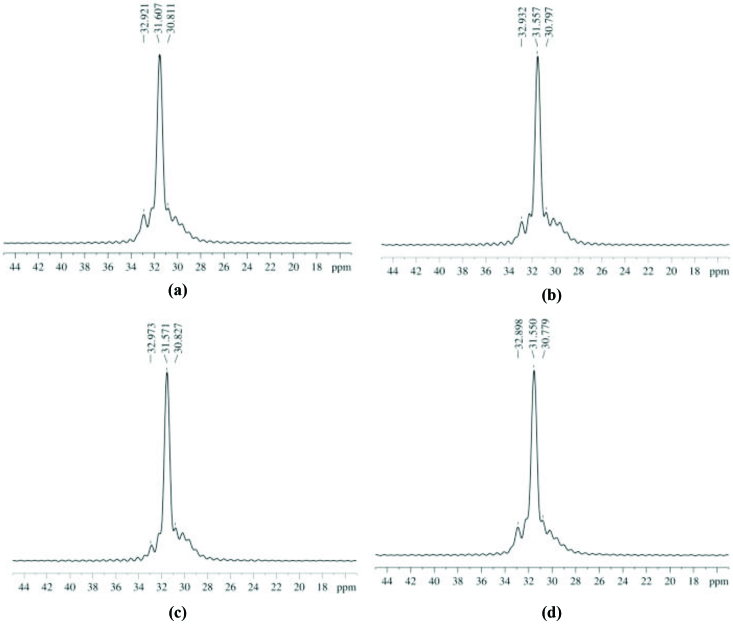
The experimental ^13^C-NMR patterns of a) Productivity (O1) b) Catalyst activity (O2) c) Molecular weight (O3) d) Crystallinity (O4).

Figure 6 demonstrates the solid state ^13^C-NMR spectra of obtained polymer samples (O1–O4). In order to observe the effect of temperature and pressure conditions on polymer morphology, NMR spectra of various samples were experimented. According to literature, polyethylene shows 3 polymorphic forms that are orthorhombic (ORC, stable as thermodynamically), triclinic, and monoclinic (MCC) [33]. While the largest peak at around 31.6 ppm is the characteristic ORC form existing in the crystalline regions, peak at 32.9 ppm is referred as MCC form of the polyethylene. The peak at 30.8 ppm shows the amorphous region of samples [34]. The 13C chemical shift behavior of the peaks are 22 similar to that of unoriented solution casted UHMWPE [35].

### 3.3. Catalyst stability for optimum conditions

**Figure 7:**
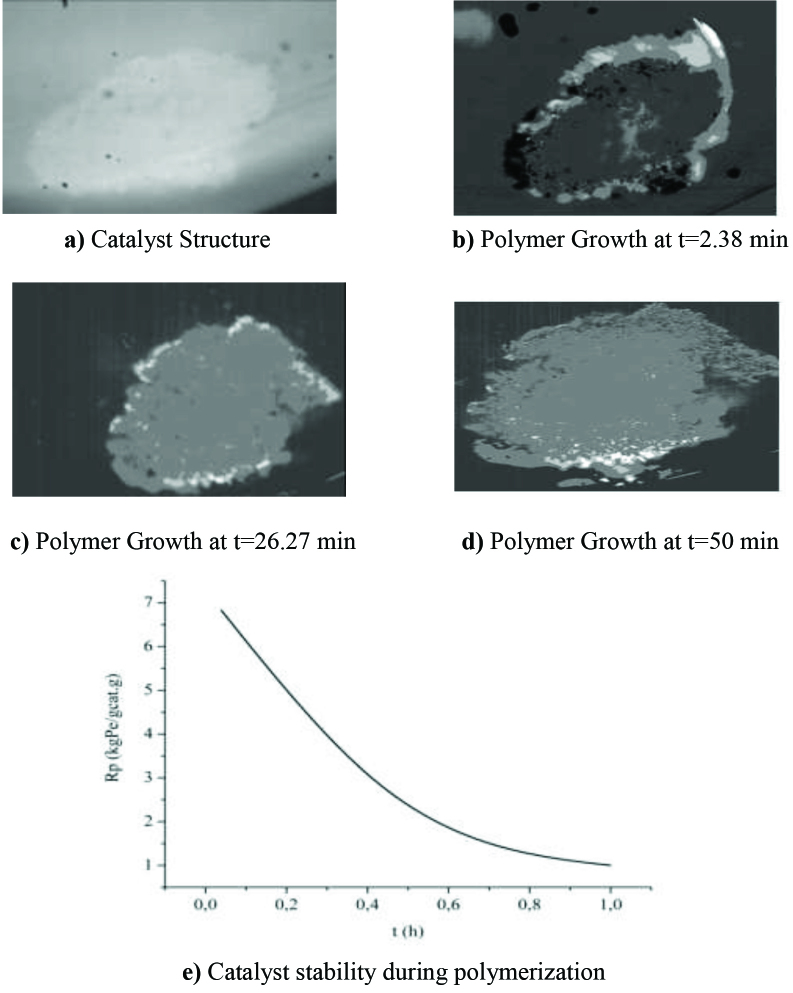
Raw and processed images obtained during experiment a) catalyst structure (raw) b) polymer particle at 2.4 min (processed) c) polymer particle at 26.3 min (processed) d) polymer particle at 50 min (processed) e) Catalyst stability during polymerization.

Ethylene polymerization consists of active sites formation, initiation, propagation and termination reactions [36]. In Figures 7a–7d, raw image of catalyst structure (a) and processed images of polymer particles (b–d) were given in accordance with polymerization time. By using Shadow Sizing program and Eq. (14–16), rate of polymerization was determined in order to observe the catalyst behavior during the experiments. Figure 7e demonstrates the polymerization time history for the polymer obtained at optimum conditions. At the initial stages, polymerization rate is high since polymerization phase (including initiation and formation) occurs during this time (until 0.6 h). After that time, activity decreases because of the diffusion limitations occurred in the catalyst due to the fragmentation, and polymer grows slowly [37].

### 3.4. Comparison with slurry systems

For the current fluidized bed runs, UHMWPE with maximum molecular weight of 6,89 x 10^6^ g moles^-1^ was produced at polymerization conditions of 45 °C, 10 bars, Al/Ti:1 and 120 min of polymerization time. With the optimum conditions determined according to Taguchi experimental design, the molecular weight of 4,17x10^6^ g moles^-1^ was successfully produced. Optimum conditions were determined as 45 °C, 7 bars, Al/Ti:1 and 90 min. It can be observed that for the slurry technology, there is a need for higher temperatures and/or higher cocatalyst-catalyst ratio to produce UHMWPE with similar molecular weights obtained in this study [38–44]. Additionally, crystallinity values of polymers and their melting temperatures show good agreements with the polymers produced by slurry technology. In their study, Zohuri et al., reported the crystallinity values were between 49% and 60% and melting temperatures were between 135–144 °C [38]. The results indicate that the gas phase produced polymers are in accordance with commercial UHMWPE [39].

This study demonstrates the possibility of gas phase process for UHMWPE, once the fluidization approach is changed accordingly, and paves the road to a new design of reactors for this process. Some of the pitfalls of traditional FBRs are related to sparged bubble dynamics. The gas dynamics determine the size of the bubbles, and the bubble dynamics are strongly sparger structure and particle size dependent. As the dynamics of the reactor starts with Geldart C type, coalescence of bubbles cause gas bypassing and channeling, which results in poor mass transfer, oscillations in heat transfer and eventually formation of hot-spots. The temperature oscillations, if it does not deactivate the catalyst right away, deactivation before the single catalyst formed UHMWPE could cause wide variation of molecular weights and a mix of UHMWPE and HDPE in the same system, which would bring separation problems. Moreover, since mass transfer in ethylene polymerization occurs in two ways: to the catalysts pores from monomer and to the amorphous regions from polymer phase, diffusion is the major transport method of ethylene polymerization [45]. Monomer diffusion coefficient of ethylene is dependent on particle morphology, temperature and polymer molecular and physical properties. At low temperatures ethylene effective diffusivity coefficient (D_*eff*_) is 10^-3^ cm^2^/s and close to the bulk diffusivity coefficient, however at higher temperatures above 90 ^o^C, D_*eff*_ is about 10^-7^ cm^2^ s^-1^ due to the clogging of the pores [46]. Eventually, oscillations in temperature would cause poor mass transfer as well.

With the current proposed system, the disadvantages of traditional FBRs can be overcome and UHMWPE homopolymers can be produced successfully.

## 4. Conclusions

In this article, a gas phase process using a mini reactor instead of conventional slurry reactors has been established to produce UHMWPE for the first time. Since traditional FBRs have problems of complex gas dynamics which can be detrimental to the process, another approach where the solids are dispersed and the gas phase forms the bulk phase is suggested. By introducing this new concept, having the solids as the dispersed phase, it is possible to remove the adverse effects of bubble dynamics, and establish perfect heat and mass transfer control, at the expense of monomer gas. Since the unreacted gas can be stored and reused, the approach gives promising ways for new design concepts in fluidized bed reactors in olefin polymerization reactions, and especially for the production UHMWPE. In order to obtain the optimum parameters for UHMWPE synthesis, Taguchi experimental design was used and parameters were determined as 7 bars, 45 °C, TEA/Ti:1 and 90 min. Higher molecular weight polymers were obtained with increasing temperature, pressure and polymerization time; however, the molecular weight decreased at higher TEA/Ti ratios. ANOVA results showed that four parameters have significant effect on the molecular weight of polyethylene. The highest molecular weight value was calculated as 6,89 x 10^6^ g moles^-1^. The results of optimum condition experiments have matched with the predicted values calculated by the confidence limits of the experimental design.
